# Impact of an electronic medical-record–embedded clinical-decision support tool on duration of antibiotics for outpatient pediatric skin and soft-tissue infections

**DOI:** 10.1017/ice.2023.232

**Published:** 2024-03

**Authors:** Elizabeth C. Lloyd, Nicholas O. Dillman, Alison C. Tribble, Lei Wu, Stephannie Seidl, Heather L. Burrows, Lindsay A. Petty

**Affiliations:** 1 Division of Pediatric Infectious Diseases, Department of Pediatrics, University of Michigan, Ann Arbor, Michigan; 2 Department of Pharmacy, University of Michigan, Ann Arbor, Michigan; 3 Quality Department, University of Michigan, Ann Arbor, Michigan; 4 Division of General Pediatrics, Department of Pediatrics, University of Michigan, Ann Arbor, Michigan; 5 Division of Infectious Diseases, Department of Internal Medicine, University of Michigan, Ann Arbor, Michigan

Up to 90% of human antibiotic use occurs in the ambulatory setting.^1,2^ In the United States, ∼50% of outpatient antibiotic prescriptions are unnecessary or inappropriate when accounting for antibiotic selection, dose, and duration.^3–7^ In addition to driving antimicrobial resistance, unnecessary antibiotic use results in increased adverse drug events and increased risk of *Clostridioides difficile* infection.^8^

Most ambulatory stewardship interventions reported in the literature have targeted respiratory infections, although skin and soft-tissue infections (SSTIs) are a common indication for outpatient antimicrobial use. We investigated the impact of an electronic medical record (EMR)–embedded clinical decision support (CDS) tool on antibiotic duration for outpatient pediatric SSTI.

## Methods

### Intervention timeline

The Michigan Medicine Ambulatory Antimicrobial Stewardship Program instituted serial interventions to improve antibiotic prescribing for outpatient pediatric SSTI. The primary intervention was a pediatric SmartSet, an EMR-embedded CDS tool used to guide therapy at the time of the visit (Epic, Epic Systems Corporation, Verona, Wisconsin).

Institutional SSTI treatment guidelines for pediatric and adult patients were developed in July 2019, followed by dedicated SSTI education for general pediatricians in May 2020. General medicine and family medicine practitioners received generalized education on the management of common infections, including SSTI. The pediatric SmartSet was developed with a general pediatrics champion and was implemented in December 2020. It includes a templated progress note and prepopulated options for guideline-concordant antibiotic prescribing based on diagnosis (impetigo, cellulitis, or abscess). In May 2021, a Tableau dashboard (Tableau software, Seattle, WA) was developed with the quality and analytics team. The dashboard displays the rate of prescriptions with an inappropriate antibiotic duration for adult and pediatric SSTI, as well as rates by clinic and provider, allowing for individual review of inappropriate cases.

### Study design, setting, and population

In this quasi-experimental, before-and after study, adult patients (aged ≥21 years) and pediatric patients (aged 2 months to <21 years) were included if they were prescribed an enteral antibiotic within 3 days of a Michigan Medicine primary care or general surgery encounter (ie, in-person, virtual, or telephone) with an *International Classification of Disease, Tenth Revision* (ICD-10) diagnosis code for impetigo, cellulitis, or cutaneous abscess. Prescriptions of <3 days or >14 days were excluded because these durations were likely not prescribed for uncomplicated SSTI. The SmartSet was implemented in December 2020. The preintervention period was July 2019 through December 2020, and the postintervention period was January 2021 through December 2021.

### Primary outcome

The primary outcome was the proportion of antibiotic prescriptions for pediatric SSTI with an inappropriate duration, defined as >7 days. Institutional SSTI guidelines, consistent with Infectious Diseases Society of America guidelines, recommend 5-day treatment courses for impetigo, cellulitis, and abscess, with extension to 7 days for slow clinical response.^9^

### Statistical analysis

The proportions of pediatric antibiotic prescriptions with an inappropriate duration were compared before and after the intervention using an interrupted time series (ITS) analysis of aggregate quarterly data.^10^ The ITS analysis used the Prais-Winsten model, which incorporates adjustment for first-order autoregressive effects. The trend in adult antibiotic duration over the study period was modeled with linear regression rather than ITS because there was no primary intervention. Analyses were performed using Stata version 13 software (StataCorp, College Station, TX).

## Results

We included 3,786 visits (948 pediatric and 2,838 adult) in the preintervention group and 2,122 visits (469 pediatric and 1,653 adult) in the postintervention group. The mean patient age was 9.9 years for pediatric patients and 55.4 years for adult patients, respectively. Overall, 84% of pediatric visits and 80% of adult visits were in person. Also, 90% of pediatric visits and 73% of adult visits were with a resident or attending physician versus an advanced practice provider. Cellulitis comprised 54% of pediatric cases and 75% of adult cases, whereas abscesses comprised 22% of pediatric cases and 25% of adult cases. The remaining 24% of pediatric cases were impetigo cases.

**Figure 1.**
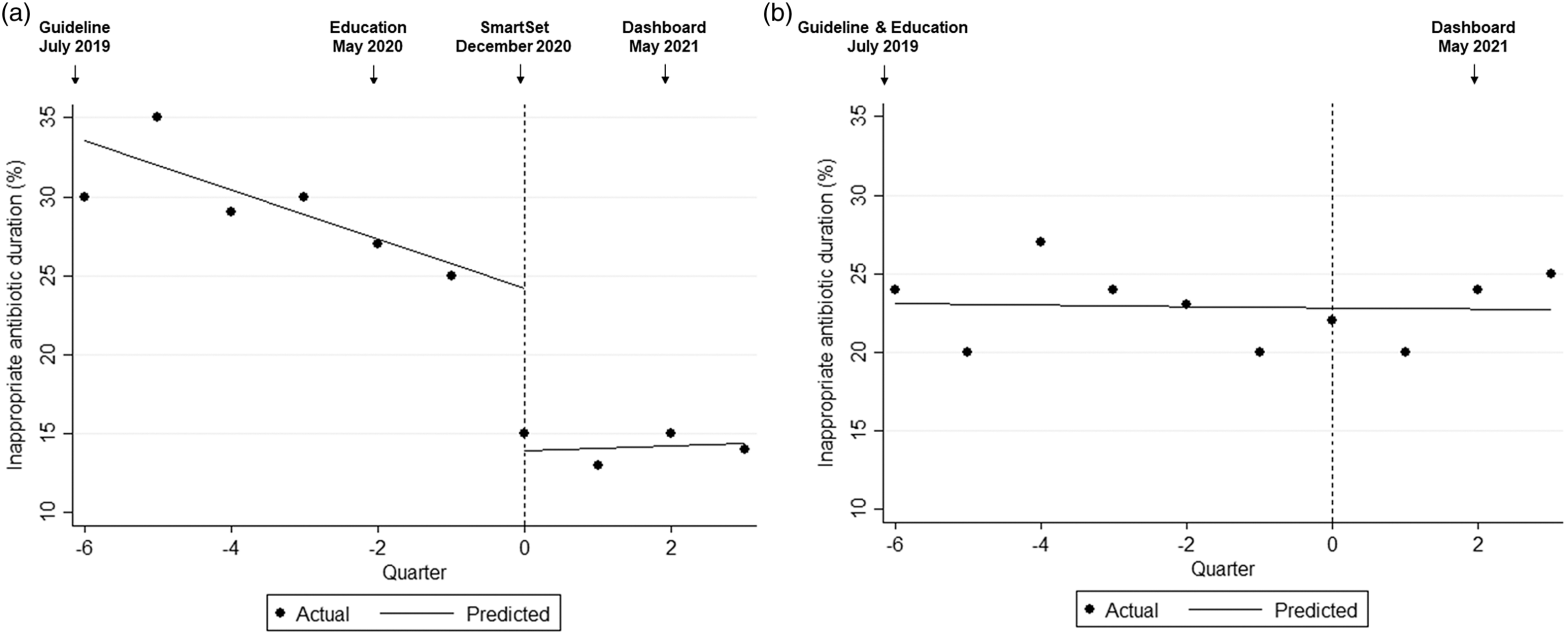
Raw and modeled percentages of inappropriate antibiotic duration for (a) pediatric and (b) adult skin and soft-tissue infection before (ie, quarters −6 to 0, encompassing Q3 2019 through Q4 2020) and after (ie, quarters 0 to 4, encompassing Q1 2021 through Q4 2021) implementation of an electronic medical record–embedded clinical-decision support tool for pediatric patients. Time is represented in 3-month intervals, with the dotted line at quarter 0 indicating Q1 2021, the first quarter after SmartSet implementation at the end of December 2020.

The SmartSet was used in 58 (12.4%) of 469 postintervention cases. The median duration of antibiotics for pediatrics was 7 days both before and after the intervention, but prescriptions with ≤5-day durations increased from 18.3% to 39.7%. An ITS analysis showed that following the release of guidelines and education to pediatric clinicians, the proportion of pediatric antibiotic prescriptions of inappropriate duration decreased by 1.6% per quarter (*P* < .01) from a high of 35% in quarter 4 of 2019. After SmartSet implementation, the proportion of prescriptions of inappropriate duration immediately decreased by 10.3% (*P* < .01), a relative decrease of 40% from the modeled percentage just prior to the intervention (Fig. 1a). After the intervention, the proportion of prescriptions of inappropriate duration remained stable at ≤15%.

For adult patients, the median antibiotic duration was 7 days, and 25.7% of prescriptions had durations of ≤5 days. The proportion of antibiotic prescriptions of inappropriate duration averaged 22.9% and did not change over the study period (*P* = .88) (Fig. 1b).

## Discussion

Implementation of an EMR-embedded CDS tool was associated with an immediate relative decrease of 40% in inappropriate antibiotic duration for outpatient pediatric SSTI. Prior to SmartSet implementation, guidelines and clinician education resulted in only a modest decline in inappropriate antibiotic duration. Subsequent passive feedback via dissemination of a Tableau dashboard sharing inappropriate prescribing rates by clinician or clinic did not result in a further reduction in inappropriate prescribing. The improvement was sustained over 12 months. SmartSet use was low, though concordant with the degree of improvement. We hypothesize that increasing SmartSet use may result in further improvement.

In contrast to the improved prescribing in pediatrics, the proportion of prescriptions of inappropriate duration for adult patients within the same health system remained unchanged over the study period. Adult providers received comparable SSTI guidelines and generalized education on treatment of common infections including SSTI but no CDS tool. These findings suggest that timely EMR nudges are associated with improved outpatient antibiotic prescribing for duration for SSTI over guidelines and education alone.
